# The Outcome of Primary Hepatic Carcinoid Tumor: A Retrospective Study Based on Propensity Score Matched Survival Analysis

**DOI:** 10.3389/fonc.2021.609397

**Published:** 2021-02-24

**Authors:** Shaotao Jiang, Huijie Wu, Rongdang Fu, Jialuo Mai, Jiyou Yao, Xuefeng Hua, Huan Chen, Jie Liu, Minqiang Lu, Ning Li

**Affiliations:** ^1^ Department of HBP SURGERY II, Guangzhou First People’s Hospital, School of Medicine, South China University of Technology, Guangzhou, China; ^2^ Department of Gynecology, The First People’s Hospital of Foshan, Foshan, China; ^3^ Department of Hepatic Surgery, The First People’s Hospital of Foshan, Affiliated Foshan Hospital of Sun Yat-sen University, Foshan, China

**Keywords:** SEER, surgery, prognosis, propensity score matching analysis, primary hepatic carcinoid tumor

## Abstract

**Background:**

Primary hepatic carcinoid tumor (PHCT) is rare and has unclear clinical characteristics and prognosis.

**Methods:**

A retrospective study using data from the SEER database for patients diagnosed with PHCT used univariate and multivariate Cox models to screen for independent prognostic factors. The outcomes of patients in the surgical and nonsurgical groups were compared, and Propensity Score Matching (PSM) analysis was used to reduce confounder bias.

**Results:**

A total of 186 PHCT patients were identified and the median survival was 65 (95% CI [43.287, 86.713]) months. Tumor size(HR = 2.493, 95% CI[1.222,5.083], p = 0.012), male(HR = 1.690, 95% CI[1.144,2.497], p = 0.008), age(HR = 2.583, 95% CI[1.697,3.930], p < 0.001), SEER stage(HR = 1.555, 95% CI[1.184,2.044], p = 0.002) and surgery(HR = 0.292, 95% CI[0.135,0.634], p = 0.002) were significantly correlated with patient prognosis. In multivariate analysis, sex(HR = 3.206, 95% CI[1.311,7.834], p = 0.011) and surgery(HR = 0.204, 95% CI[0.043,0.966], p = 0.0045) were independent predictors of patient prognosis. Females are potentially susceptible to PHCT but have a better prognosis. With consistent baseline data, surgical patients have a better prognosis.

**Conclusions:**

PHCT is uncommon and survival time is longer than that of other primary liver cancers. We found that none-surgery was potentially independent risk factors for poor prognosis.

## Introduction

Since the concept of carcinoid was proposed in 1907, most carcinoid tumors are thought to be malignant tumors originating from neuroendocrine cells, and commonly occurring in the gastrointestinal tract (1). The incidence of carcinoid is increasing (2). The liver is a common organ for gastrointestinal carcinoid metastases (3), however, primary hepatic carcinoid tumor (PHCT) was rarely seen (4). Besides, the origin of PHCT is complex and remains unclear. Previous literature indicates that neuroendocrine cells distributed in the bile duct are stimulated by biliary tract inflammation, which may be an important factor in the occurrence of carcinoid (5, 6). At the same time, some studies have shown that the original stem cells of the liver can also turn malignant into carcinoids (7).

Gastrointestinal carcinoid and pulmonary carcinoid patients are mainly treated with complete tumor resection, which can achieve good long-term prognosis (2, 8, 9). Surgery is performed to preserve as much normal tissue as possible while excising the margins of the carcinoid. Previous case reports and small sample retrospective studies have also shown that PHCT patients may achieve longer overall survival after surgery (10, 11). However, early diagnosis of PHCT is difficult, requiring active exclusion of metastases from other organs and often requiring pathological confirmation. Patients with advanced PHCT have a poor prognosis (12) and more data are needed to support whether they can benefit from surgery. Besides, tumor size and stage are important indicators affecting prognosis and whether patients can receive surgical treatment. Due to most of the existing studies are small samples or case reports (13), the selection criteria for surgical patients are unclear.

Therefore, we used the Surveillance, Epidemiology, and End Results (SEER) database to assess cancer specific survival (CSS) in patients with PHCT. Factors related to CSS were screened to identify independent factors affecting the prognosis of PTCH patients. The surgical benefit to PHCT patients was assessed after propensity score matched (PSM) with the nonsurgical group.

## Materials and Methods

### Patients

This study was a retrospective analysis, with all data from the SEER database from 1975 to 2016. Patients’ demographic and clinical data were downloaded using SEER*Stat software. According to the SEER database coding manual, all patients with Histologic Type, coded by the International Classification of Diseases for Oncology (ICD-O-3) of 8240/3 Carcinoid tumor and the primary site is limited to the liver were included. Patients without definite survival months or SEER cause-specific death classification were excluded. Patients with two or more malignancies at the time of diagnosis of PHCT were also excluded. In addition, 8170/3 Hepatocellular carcinoma were also included in the study as a comparison cohort. All authors have applied for and obtained permission to use the database from SEER’s official website. The data are public and do not involve the privacy of patients, so the review and consent of the ethics committee are not required.

### Definition of Variables

A detailed definition of all variables in this study is available in the SEER database manual. Histologic stages associated with the degree of the tumor invasion, Localized refers to limited to primary organs, Regional refers to invade beyond primary organs or local lymph node invasion, and Distant refers to metastatic lesions found in the distance. Grade was related to the degree of differentiation of tumor cells, which were well differentiated, moderately differentiated, poorly differentiated and undifferentiated, respectively. Local destruction in the type of surgical treatment involved photodynamic therapy, electrocautery, cryosurgery, laser, alcohol injection and Heat-Radio-Frequency ablation.

### Statistical Analysis

Continuous variables such as age and tumor size were converted into categorical variables by the X-tile software (14) with the optimal cut off value through enumeration method. For univariate analysis, the Cox regression model was used to assess hazard ratio (HR) and 95% confidence intervals (CI). Cancer specific mortality was used as the event. Variables with p < 0.05 were included in the multivariate analysis to predict independent prognosis factors, and their CSS curve was performed using the Kaplan-Meier method. Then, all significant variables in univariate analysis of PHCT were extracted and matched with HCC according to the PSM principle. Finally, we performed 1:1 PSM for patients with or without surgical treatment and their prognoses were compared too. For patients with liver tumors with different pathological type, we used 1:4 PSM to compare their prognosis. Propensity score (PS) is calculated using logit model. PS for each patient was obtained from a multivariable Cox regression model based on patient characteristics. We chose the caliper value at 20% of the standard deviation of the PS value converted by logit model, because it is commonly used. R (version 3.5.1, https://www.r-project.org/) and IBM SPSS (version 25) were used for statistical analysis.

## Results

### Clinicopathological Characteristics of Patients With Primary Hepatic Carcinoid Tumor

**Figure 1:**
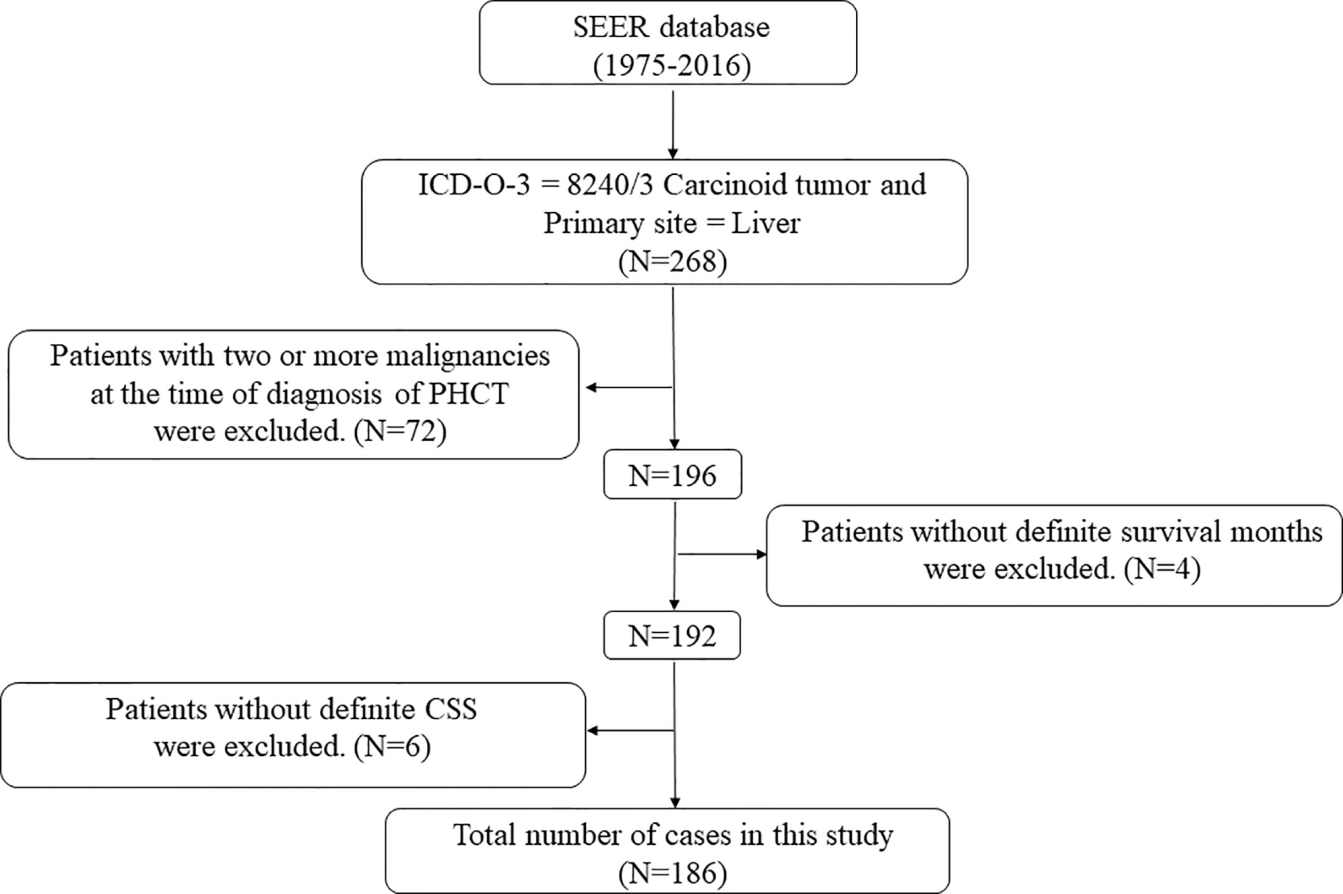
The flow charts. The screening process of PHCT patients in this study.

The screening process of PHCT patients in this study is shown in **Figure 1**. Of the 268 patients with PHCT, 72 patients with other malignancies and 10 patients with follow-up data loss were excluded, and 186 patients finally met the criteria. The baseline characteristics of these patients are shown in **Table 1**. About demographic data, 83.3% (n = 155) of the patients were white, and more than half of them are females (n = 100, 53.8%). In this study, patients were divided by diagnosis age into ≤62 groups and >62 groups, with numbers of 83 (44.6%) and 103 (55.4%), respectively. Similarly, 37 patients (19.9%) had tumors ≤55 mm, 35 patients (18.8%) had tumors >55mm, and the rest had unknown tumor sizes. Further, the histologic stage is an indicator to assess tumor invasion. The number of patients with Localized is the largest (n = 68, 36.6%), followed by distant (n = 37, 19.9%), and 26.3% patients(n = 49) with unknown histologic stage. Grade information refers to the degree of tumor differentiation. Carcinoid tumor of 16.7% patients (n = 31) were well differentiated and 2.2% (n = 4) were poorly differentiated. However, Grade information of more than three-quarters of patients (n = 140,75.3%) was unknown. Finally, surgery was performed in 14.5% of patients, including destruction of local tumor lesions (n = 4, 2.2%), or wedge resection (n = 11, 5.9%), or lobectomy (n = 12, 6.5%), and more than half of the patients (n = 137,73.7%) did not undergo surgery.

**Table 1:** Baseline characteristics of 186 patients with primary hepatic carcinoid tumor.

Variables		n (186)	100%
Race			
	Black	25	13.4
	Other	6	3.3
	White	155	83.3
Sex			
	Female	100	53.8
	Male	86	46.2
Age			
	≤62	83	44.6
	>62	103	55.4
Size(mm)			
	≤55	37	19.9
	>55	35	18.8
	Unknown	114	61.3
Stage			
	Localized	68	36.6
	Regional	32	17.2
	Distant	37	19.9
	Unknown	49	26.3
Grade			
	I	31	16.7
	II	9	4.8
	III	4	2.2
	IV	2	1.1
	Unknown	140	75.3
Surgery			
	None	137	73.7
	Local destruction	4	2.2
	Wedge resection	11	5.9
	Lobectomy	12	6.5
	Unknown	22	11.8

### Univariate and Multivariate Analysis of Cancer Specific Survival in Patients With Primary Hepatic Carcinoid Tumor

The Cox hazard ratio model was performed to screen prognostic factors for PHCT patients. For univariate analysis, tumor diameter > 55 mm (HR = 2.493, 95% CI[1.222,5.083], p = 0.012), male (HR = 1.690, 95% CI[1.144,2.497], p = 0.008), age > 62 (HR = 2.583, 95% CI[1.697,3.930], p < 0.001), regional stage (HR = 1.974, 95% CI[1.105,3.527], p = 0.022) and distant stage (HR = 2.393, 95% CI[1.362,4.205], p = 0.002) were found associated with higher risk. Besides, patients who underwent surgery showed lower risk of mortality than those who did not (HR = 0.292, 95% CI[0.135,0.634], p = 0.002). Those variables were further studied by multivariate analysis. To sum that, male (HR = 3.206, 95% CI[1.311,7.834], p = 0.011) and surgery (HR = 0.204, 95% CI[0.043,0.966], p = 0.0045), respectively, presented as an independent factor of primary hepatic carcinoid tumor patients (**Table 2**).

**Table 2:** Univariate and multivariate analysis of cancer specific survival in patients with primary hepatic carcinoid tumor.

Variables		Univariate analysis		Multivariate analysis	
		HR (95CI)	P-value	HR (95CI)	P-value
Sex				3.206(1.311,7.837)	0.011
	Female	Ref	–		
	Male	1.690(1.144,2.497)	0.008		
Age				1.894(0.835,4.294)	0.126
	≤62	Ref	–		
	>62	2.583(1.697,3.930)	<0.001		
Race					
	White	Ref	–		
	Other	0.912(0.497,1.673)	0.765		
Size(mm)				1.39(0.637,3.032)	0.408
	<55	Ref	–		
	>56	2.493(1.222,5.083)	0.012		
Grade					
	I/II	Ref	–		
	III/IV	1.063(0.335,3.372)	0.917		
SEER stage				1.401(0.872,2.249)	0.163
	Localized	Ref	–		
	Regional	1.974(1.105,3.527)	0.022		
	Distant	2.393(1.362,4.205)	0.002		
Surgery				0.204(0.043,0.966)	0.045
	None	Ref	–		
	Yes	0.292(0.135,0.634)	0.002		

### Patients Cancer Specific Survival

**Figure 2:**
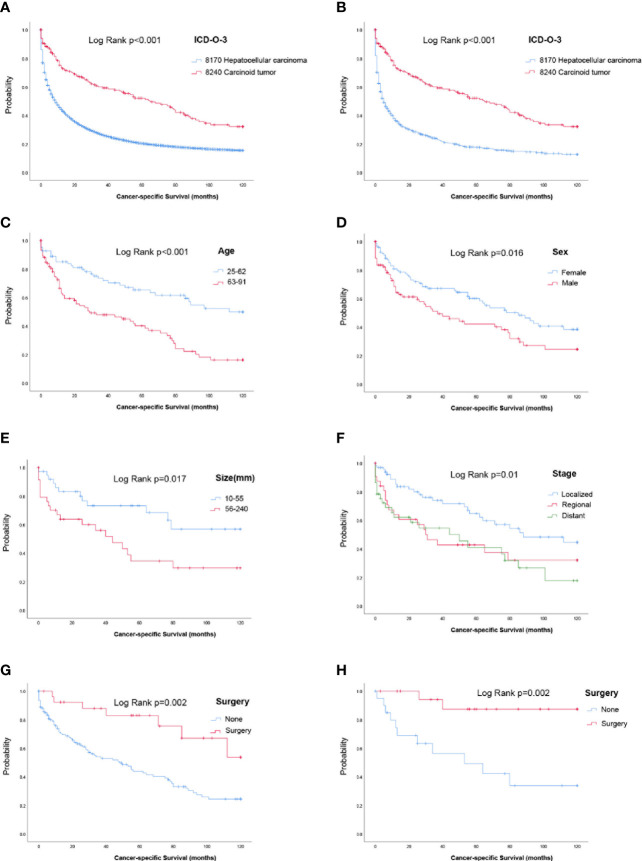
Survival analysis. **(A)** Survival analysis for primary hepatic carcinoid tumors or hepatocellular carcinoma patients before propensity score matching. **(B)** Survival analysis for primary hepatic carcinoid tumor or hepatocellular carcinoma patients after propensity score matching. **(C–G)** Cancer specific survival curve for carcinoid tumor patients based on age, sex, tumor size, stage, and surgery. **(H)** Cancer-specific survival curve for carcinoid tumor patients who underwent surgery after propensity score matching.

Survival analysis was performed in 186 PHCT patients and 88159 HCC patients with certain cancer specific survival status and time. Summarily, the CSS for patients with PHCT was better than those who with HCC (**Figure 2A**). In detail, median CSS in PHCT and HCC patients was 65 (95% CI [43.287, 86.713]) and 9 (95% CI [8.809, 9.191]) months, respectively. Furthermore, to describe more objectively the difference between PHCT and HCC, a 1:4 PSM analysis including variable of age, tumor size, sex, stage, and surgery was performed. Finally, 186 PHCT patients were matched with 744 HCC patients. There were no significant differences were observed between them (**Table 3**). As shown in **Figure 2B**, CSS for patients with carcinoid tumor were still better than those who with HCC (p < 0.001).

**Table 3:** Characteristics of patients before and after propensity score matching.

		Before PSM			After PSM	
		PHCT	HCC	P	HCC	P
**n**		186	88176		744	
**Age (mean (SD))**		63.06 (14.10)	63.15 (11.74)	0.917	64.03 (13.30)	0.38
**Sex (%)**	Female	100 (53.8)	20714 (23.5)	<0.001	389 (52.3)	0.78
	Male	86 (46.2)	67462 (76.5)		355 (47.7)	
**Stage (%)**	Localized	68 (36.6)	39090 (44.3)	<0.001	269 (36.2)	0.996
	Regional	32 (17.2)	23793 (27.0)		132 (17.7)	
	Distant	37 (19.9)	14383 (16.3)		144 (19.4)	
	Unknown	49 (26.3)	10910 (12.4)		199 (26.7)	
**Surgery (%)**	None	137 (73.7)	63082 (71.5)	<0.001	559 (75.1)	0.288
	Local destruction	4 (2.2)	7359 (8.3)		27 (3.6)	
	Wedge resection	11 (5.9)	3237 (3.7)		25 (3.4)	
	Lobectomy	12 (6.5)	2649 (3.0)		39 (5.2)	
	Transplant	0 (0.0)	3987 (4.5)		9 (1.2)	
	Unknown	22 (11.8)	7845 (8.9)		85 (11.4)	
**Size (%)mm**	0-30	18 (9.7)	18153 (20.6)	<0.001	58 (7.8)	0.945
	31-55	19 (10.2)	17728 (20.1)		82 (11.0)	
	56-120	22 (11.8)	19933 (22.6)		81 (10.9)	
	121-240	13 (7.0)	5577 (6.3)		51 (6.9)	
	240+	0 (0.0)	208 (0.2)		1 (0.1)	
	Unknown	114 (61.3)	26577 (30.1)		471 (63.3)	

For PHCT, patients with age younger than 62 had longer CSS time (5years CSS rate, 65.4 vs. 40.2%) (**Figure 2C**). Besides, female patients had longer CSS time, with 60.1% 5 years CSS rate, compared with 42.1% for males (**Figure 2D**). For patients with tumors size bigger than 56 mm, their 5 years CSS rate was only 34.4%, and it was 73.3% for those who less than 55 mm (**Figure 2E**). Similarly, for the localized, regional, and distant histologic stage, the 5 years CSS rate was 64.7%, 42.8%, and 41.0%, respectively (**Figure 2F**). Patients who underwent surgery had a better prognosis, with a 5 years CSS rate of 83%, and 43.8% for those who had no surgery (**Figure 2G**).

### Survival Analysis of Surgery for Primary Hepatic Carcinoid Tumor Patients

Previous analyses have shown that PHCT patients who have undergone surgery have a longer CSS month. Moreover, age, sex, histologic stage, and tumor size were also significantly correlated with the prognosis of patients. Of the 186 patients, 63 (surgical = 21, nonsurgical = 42) had known age, tumor size, sex, histologic stage, and surgery. Furthermore, there were significant differences in age (p = 0.016), sex (p = 0.025), and histologic stage (p = 0.002) between the surgical group and the nonsurgical group. To avoid the additional impact of these variables on patient survival, we performed 1:1 PSM for age, tumor size, sex, and stage in these two groups. Age and tumor size were matched as continuous variables. After PSM, there was no significant difference in clinical baseline data between the matched patients in the surgical group and the nonsurgical group, as shown in **Table 4**. PHCT patients underwent surgery still showed longer CSS (p = 0.002, **Figure 2H**).

**Table 4:** Characteristics of surgical and nonsurgical patients before and after propensity score matching.

	Before PSM				After PSM	
	Level	Surgery	Nonesurgery	p	Nonesurgery	p
n		21	42		21	
Age (mean (SD))		53(16.29)	62.62(13.5)	0.016	61.38(12.48)	0.069
Size (mean (SD))		76.33(68.56)	77.74(47.71)	0.925	68.52(38.28)	0.651
Sex (%)	Female	14(66.7%)	14(33.3%)	0.025	9(42.9%)	0.215
	Male	7(33.3%)	28(66.7%)		12(57.1%)	
Stage (%)	Localized	17(81%)	16(38.1%)	0.002	16(76.2%)	1
	Regional	4(19%)	13(31%)		5(23.8%)	
	Distant	0	13(31%)		0	

## Discussion

Classification by original site, carcinoid tumor most often occurs in gastrointestinal organs such as the small intestine (1). Besides, liver metastases from gastrointestinal carcinoid tumors are common. However, PHCT is a rare type of malignant liver tumor, and the original cell is unclear (13). Previously, most of the existing studies are small samples or case report s (13, 15–19) and Yao JC et al. summarized the incidence and characteristics of 43 PHCT cases in the SEER database from 1973 to 1999 (4). However, twenty years have been passed, the clinicopathologic features, prognostic factors, and overall prognosis of PHCT are still unclear. As the number of cases in the SEER database increased, we further explored the clinical characteristics and prognosis of PHCT, and to identify independent factors that affect prognosis. To the best of our knowledge, this study has the largest number of patients with HCT so far.

Gravante G et al. reviewed 69 cases with a median survival rate of 31 months (13). In our study, however, the median survival estimates for 186 PHCT patients was 65 months. This can be caused by a variety of factors. First, we included a much larger sample than previous reviews. Secondly, scattered cases reported may be due to the insufficient local economic or medical level or other reasons, leading to untimely diagnosis, resulting in a poor prognosis (12). The SEER database includes cases from states in the United States with higher medical standards. Finally, differences in the year of diagnosis also contribute to these.

In this study, PHCT has the best prognosis compared to HCC and cholangiocarcinoma, which are two common types of liver cancer. In morbidity, HCC and cholangiocarcinoma are much higher than PHCT. Besides, in the univariate Cox regression model, we found that age, sex, tumor size, histologic stage, and surgery were significant factors affecting the prognosis of PHCT patients. Previous studies have also shown that these variables are significantly associated with the prognosis of HCC (20) and cholangiocarcinoma (21). Therefore, to better remove the influence of confounders and compare the prognosis of these three pathological types of liver cancer, we performed PSM with PHCT as a reference. As a result, PHCT showed a better prognosis both before and after PSM matching.

Previous literature has reported higher rates of PHCT in females than in males (22), and our results are consistent with this (53.8 vs. 46.2%). Furthermore, our results confirm that females have a better prognosis (median survival months 85 vs 37). After univariate and multivariate analyses, sex was an independent factor affecting PHCT prognosis. Sex is also an important prognostic factor in other carcinoid tumors such as bronchopulmonary carcinoid (23). Other factors, such as tumor size, age, and histologic stage, were also found to be significantly correlated with patient prognosis in the univariate analysis. However, there was no statistical significance in the multivariate analysis. Tumor size reflects the proliferation capacity and progression of the tumor. The histologic stage is an indicator to assess the degree of metastasis and invasion of the tumor. In our study, there was no significant difference in prognosis between regional and distant patients. Patients with localized have the longest CSS months and the largest number of PHCT patients. Regional or distant patients mean that the tumor has invaded surrounding tissues, or lymph nodes, or distant organs, which is undoubtedly an important factor in the poor prognosis of tumor patients (24, 25).

PHCT patients are mainly treated with complete tumor resection, which can achieve good long-term prognosis (10, 11). Local excision and systemic control of the tumor are options for maintaining a high quality of life. In the absence of local tissue invasion and metastasis of the tumor, surgical resection should be the first choice. In our study, 28 patients who underwent surgery had longer CSS months than those who did not. Simply dividing patients into surgical and nonsurgical groups is not rigorous, because there are patients with bigger size tumors, advanced stage, and older age, and they are not suitable for surgery. Their poor prognosis was clear, regardless of whether they had surgery or not. Therefore, we performed a PSM analysis of surgical and nonsurgical patients by age, tumor size, sex, and histologic stage variables. PSM reduces the effect of treatment selection bias on the outcome by matching baseline data (26). After PSM, there was no statistical difference in these baseline data between patients in the surgical and nonsurgical groups. The prognosis of patients in the surgical group was still better than that in the nonsurgical group.

As with other studies analyzing the SEER database (2, 4), our results also have some limitation. First, there is a lack of data in some patient variables, which potentially lead to biased results. To retain enough sample size, we included patients with a small number of missing variables in the analysis. Second, we do not know what treatment the patient has received other than surgery, so we cannot assess the effect of other treatments on the prognosis. To make up for these inevitable limitation in register-based studies, PSM analysis is adopted in statistical methods of this study. More cases and prospective studies need to be included in further exploration.

## Conclusions

In general, we used the SEER database to find that the prognosis of PHCT was better than that of common liver tumors such as HCC and cholangiocarcinoma. Tumor size, sex, age, histologic stage, and surgery were significantly correlated with patient prognosis. Sex and surgery were independent predictors of patient prognosis. Females are potentially susceptible to PHCT and have a better prognosis. With consistent baseline data, surgical patients have a better prognosis.

## Data Availability Statement

Publicly available data sets were analyzed in this study. These data can be found here: SEER database.

## Author Contributions

SJ: Conceptualization, statistical analysis, and writing original draft. HW: Statistical analysis, visualization, and software. RF: Software and investigation. JM, JY, XH, HC, and JL: Edited, coordinated, and provided important suggestions. ML: Supervision, writing (review and editing), and project administration. NL: Supervision, writing (review and editing), and project administration. All authors contributed to the article and approved the submitted version.

## Funding

This work was supported by National Natural Science Foundation of China [81400679], Guangdong Natural Science Foundation [2014A030310067], Guangzhou Science and Technology Programs [202002030387, 201704020153], Special fund of Foshan Summit plan [2019D006] and Foundation of Basic and Applied Basic Research of Guangdong Province [2020A1515110073].

## Conflict of Interest

The authors declare that the research was conducted in the absence of any commercial or financial relationships that could be construed as a potential conflict of interest.
